# Skeletal muscle biochemical origin of exercise intensity domains and their relation to whole-body V̇O_2_ kinetics

**DOI:** 10.1042/BSR20220798

**Published:** 2022-08-09

**Authors:** Bernard Korzeniewski, Harry B. Rossiter

**Affiliations:** 1 BioSimulation Center, Kraków, Poland; 2 Pulmonary and Critical Care Physiology and Medicine, The Lundquist Institute for Biomedical Innovation at Harbor-UCLA Medical Center, Torrance, CA 90254, U.S.A.

**Keywords:** blood lactate, exercise intensity, inorganic phosphate, muscle fatigue, oxygen consumption, skeletal muscle

## Abstract

This article presents the biochemical intra-skeletal-muscle basis of exercise intensity domains: moderate (M), heavy (H), very heavy (VH) and severe (S). Threshold origins are mediated by a ‘P_i_ double-threshold’ mechanism of muscle fatigue, which assumes (1) additional ATP usage, underlying muscle V̇O_2_ and metabolite slow components, is initiated when inorganic phosphate (P_i_) exceeds a critical value (Pi_crit_); (2) exercise is terminated because of fatigue, when P_i_ reaches a peak value (Pi_peak_); and (3) the P_i_ increase and additional ATP usage increase mutually stimulate each other forming a positive feedback. M/H and H/VH borders are defined by P_i_ on-kinetics in relation to Pi_crit_ and Pi_peak_. The values of the ATP usage activity, proportional to power output (PO), for the M/H, H/VH and VH/S borders are lowest in untrained muscle and highest in well-trained muscle. The metabolic range between the M/H and H/VH border (or ‘H space’) decreases with muscle training, while the difference between the H/VH and VH/S border (or ‘VH space’) is only weakly dependent on training status. The absolute magnitude of the muscle V̇O_2_ slow-component, absent in M exercise, rises gradually with PO to a maximal value in H exercise, and then decreases with PO in VH and S exercise. Simulations of untrained, physically active and well-trained muscle demonstrate that the muscle M/H border need not be identical to the whole-body M/H border determined from pulmonary V̇O_2_ on-kinetics and blood lactate, while suggesting that the biochemical origins of the H/VH border reside within skeletal muscle and correspond to whole-body critical power.

## Introduction

Skeletal muscle metabolic flux (flow of metabolites through the bioenergetic system, especially ATP turnover) can vary over 100-fold between resting and maximal exercise conditions. This continuous spectrum is divided into several ranges, or intensity domains, that differ qualitatively by their biochemical and kinetic behaviors. Three main classifications of exercise intensity domains in humans have been postulated in the literature (see [1,2] for review), which are determined from whole-body responses, typically in terms of pulmonary V̇O_2_ and blood lactate on-kinetics. The simplest system involves moderate (M), heavy (H) and severe (S) intensity domains.

The M exercise intensity domain is located below the lactate threshold (LT) or gas exchange threshold (GET), and the pulmonary V̇O_2_ on-kinetics comprise only cardiodynamic (phase I) and fundamental (phase II) components. In the muscle, M exercise is also characterized by an initial delay (‘lag phase’) in the V̇O_2_ on-kinetics, analogous, but mechanistically distinct, to phase I, and fundamental (phase II) components. Muscle and pulmonary phase II V̇O_2_ on-kinetics increase approximately exponentially and reach a steady-state (plateau) after approximately 2–3 min. No slow component of the V̇O_2_ on-kinetics is present. Blood lactate (L^−^) initially increases slightly above the resting level but returns to or below the resting value after a few minutes. In the steady state of M exercise, pulmonary V̇CO_2_/V̇O_2_ stabilizes at or below 1.0 [1–3].

The H exercise intensity domain comprises power outputs (POs) and metabolic fluxes (including V̇O_2_) between LT/GET and critical power (CP, the asymptote of the power–duration curve, see [4,5]). Here, the slow component of the V̇O_2_ on-kinetics appears 1.5–2 min after the onset of exercise, which is superimposed on the primary phase II on-kinetics. However, after some time (typically, 15–20 min) V̇O_2_ stabilizes at a level below V̇O_2max_, but above the level expected from phase II on-kinetics and exercise can be well sustained. Blood lactate (L^−^) also initially rises and then stabilizes at a value above resting. V̇CO_2_/V̇O_2_ transiently increases and slowly declines over ∼15–20 min to a value approximately equal to 1.0.

Finally, the S exercise intensity domain is characterized by the presence of a V̇O_2_ on-kinetics slow component that is not able to stabilize, causing V̇O_2_ to increase continuously until exercise is voluntarily terminated, or when it reaches V̇O_2max_. S intensity exercise is associated with progressive loss of efficiency related to fatigue, which continues until termination or intolerance. Blood L^−^ increases continuously throughout S intensity exercise and V̇CO_2_/V̇O_2_ increases abruptly followed by a slow decline but without achieving stability and remains above 1.0 at termination.

Two other exercise intensity domain classification schemes constitute a modification of the above classification. Some define extreme (E) exercise intensity from the greatest PO for which V̇O_2_ is able to reach V̇O_2max_; exercise in the E domain is therefore characterized by task failure from fatigue prior to reaching V̇O_2max_ [6]. E intensity exercise is typically limited to less than approximately 2 min in duration. Another modification was proposed by Whipp [1,3,7], who split the S exercise domain into very heavy (VH) and severe (S) on the basis of whether or not the primary component of the V̇O_2_ on-kinetics is predicted to project below (VH domain) or above (S domain) V̇O_2max_.

The mechanism(s) that cause exercise termination at (or below) V̇O_2max_ remain uncertain [8]. Muscle fatigue can lead to termination of exercise [9,10]. Fatigue is related to a fall in the efficiency of the skeletal muscle bioenergetic system [11]. Recently, the ‘P_i_ double-threshold’ mechanism of muscle fatigue has been proposed to help explain observed bioenergetics system behaviors [12,13]. This mechanism is based on three assumptions: (1) the additional ATP usage, which underlies the slow component of V̇O_2_ and metabolite on-kinetics, is initiated when P_i_ exceeds a certain critical value, termed Pi_crit_ [12]; (2) muscle work is terminated because of fatigue when P_i_ reaches another, higher, peak value (Pi_peak_) [14]; and (3) P_i_ increase and additional ATP usage increase mutually stimulate each other, thus forming a self-driving positive feedback mechanism [12]. This latter assumption ultimately causes P_i_ to reach Pi_peak_ (and V̇O_2_ to reach V̇O_2max_) and exercise termination because of fatigue. This mechanism is able to generate many various, apparently unrelated, muscle system properties: changes over time of several variables including muscle V̇O_2_, cytosolic ADP, pH, PCr and P_i_ during rest-to-work transition in skeletal muscle; the end-exercise constancy of these variables at different power outputs above CP; the hyperbolic shape of the power–duration curve with CP as an asymptote; and the hypoxia/hyperoxia-induced decrease/increase in CP and V̇O_2max_, and increase/decrease of *t*_0.63_ [12].

In addition, the ‘P_i_ double-threshold’ mechanism is able to account for training-induced changes in V̇O_2max_, CP and V̇O_2_ on-kinetics (shortening of *t*_0.63_, decrease of the slow component), provided that muscle training causes an increase in OXPHOS activity and decrease in Pi_peak_ [13]. The ‘P_i_ double-threshold’ mechanism is also able to account for observed effects on muscle bioenergetic responses and exercise tolerance in patients with mitochondrial and nuclear DNA mutations causing deficiencies in OXPHOS [15] and the effect of training in such patients [16].

This theoretical study aims to identify intramuscular origins of whole-body exercise intensity domains. We use the intensity domain terminology of [3] (i.e. M, H, VH and S) to define exercise intensity domains at the skeletal muscle metabolism level and to relate these to intensity domains defined at the whole-body level in terms of pulmonary V̇O_2_ and blood L^−^ kinetics. In other words, we aim to link skeletal muscle biochemical/molecular events to physiologic responses during rest-to-work transitions and development of muscle fatigue. We define the muscle exercise intensity domains in terms of the ‘P_i_ double-threshold’ mechanism of muscle fatigue, involving the P_i_ on-kinetics, Pi_crit_ and Pi_peak_, and postulate that whole-body exercise intensity domains originate primarily at the molecular level. As events defining whole-body intensity domains are influenced by extra-muscular events (e.g. blood flow distribution, lactate clearance and oxygen consumption by tissues other than working muscles), we investigate whether borders between the M and H domains are similar at the muscle and whole-body levels.

## Theoretical results

**Figure 1:**
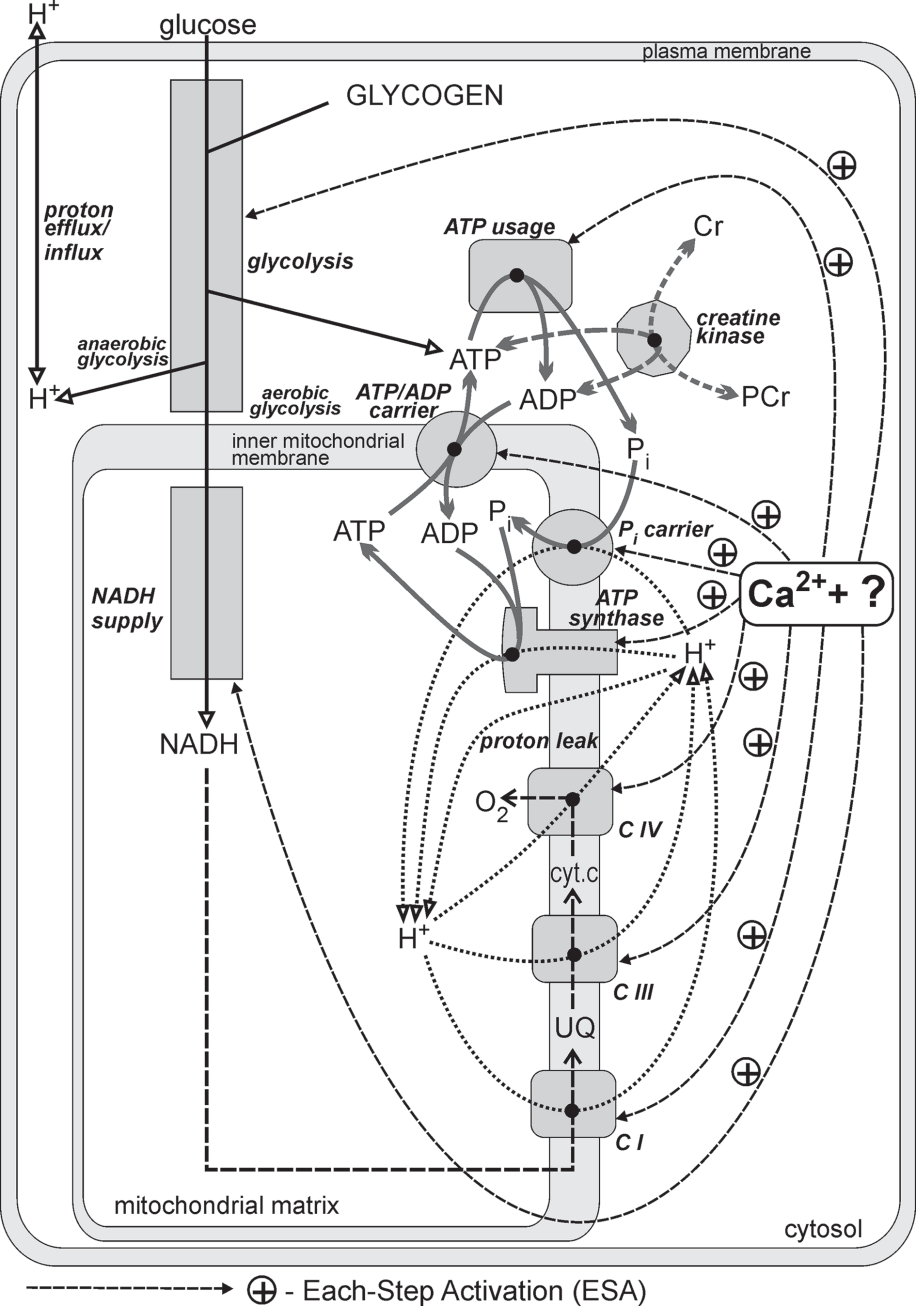
Simplified scheme of the bioenergetic system in the skeletal muscle cell The elements of the system that are explicitly taken into account within the computer model used are shown. Essentially, all elements of the system are directly activated by some mechanism involving cytosolic Ca^2+^ (inner mitochondrial membrane OXPHOS complexes, malate-aspartate shuttle and glycolysis) and mitochondrial Ca^2+^ (NADH supply). The question mark (‘?’) indicates some still undetermined factor/mechanism cooperating with Ca^2+^, for instance calmodulin-like protein ‘presenting’ Ca^2+^ to enzymes/carriers and/or protein phosphorylation. CI, CIII, CIV, complexes I, III and IV of the respiratory chain, respectively; cyt.c, cytochrome c; UQ, ubiquinone.

The scheme of the bioenergetic system, showing the elements accounted for explicitly within the model used in this study, is presented in Figure 1.

**Figure 2:**
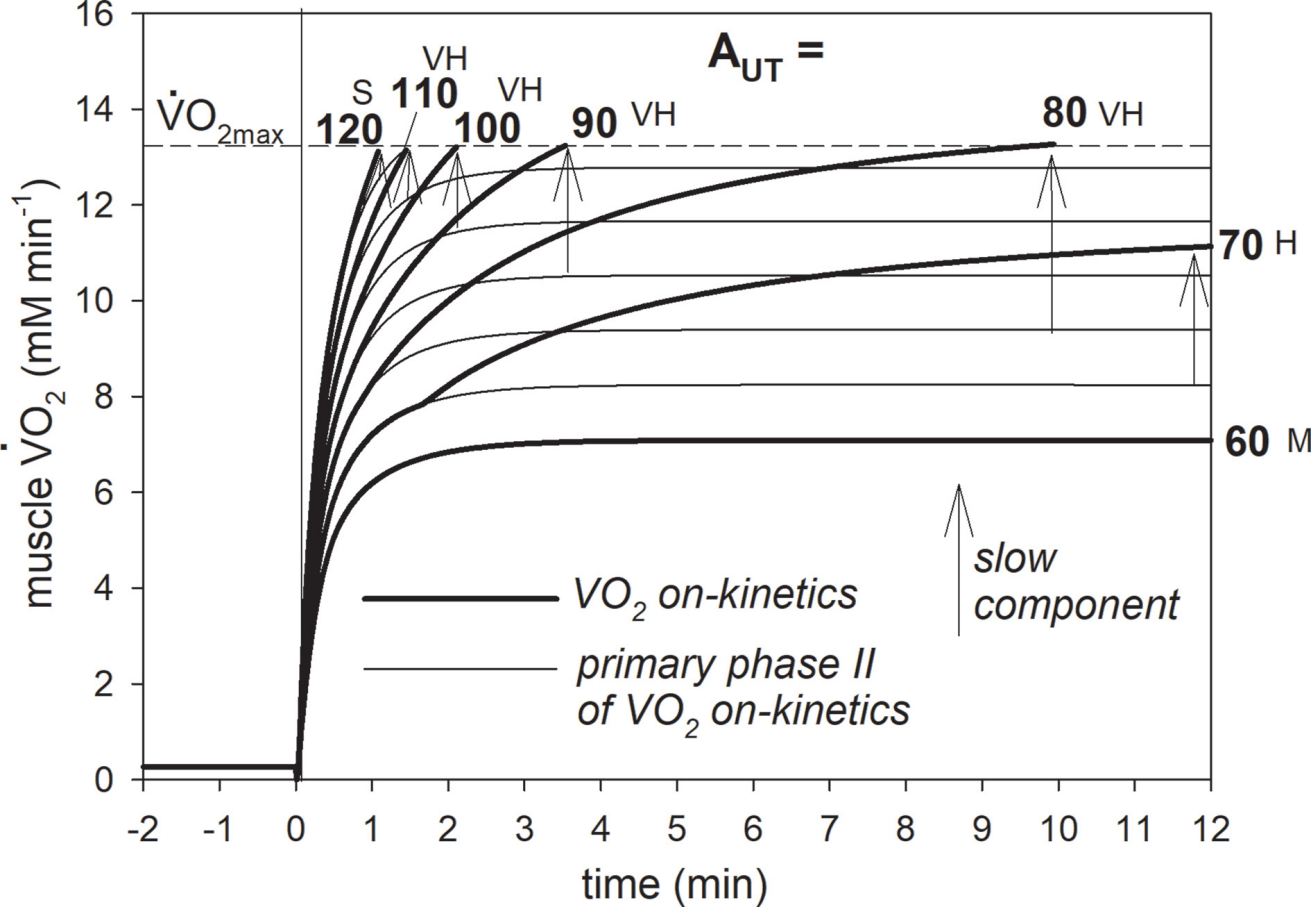
Simulated muscle V̇O_2_ on-kinetics at different ATP usage activities (A_UT_, proportional to PO) Different exercise intensity domains are present: M (A_UT_ = 60), H (A_UT_ = 70), VH (A_UT_ = 80, 90, 100, 110), S (A_UT_ = 120). The primary phase II of the V̇O_2_ on-kinetics and the magnitude of the slow component of the V̇O_2_ on-kinetics for particular exercise intensities are shown. The figure is truncated at 12 min for clarity.

Increasing ATP usage activity (A_UT_, analogous to PO) affected significantly the V̇O_2_ on-kinetics. This is demonstrated in results from the default simulation of physically active muscle in Figure 2. The steady-state V̇O_2_ of the primary phase II of the muscle V̇O_2_ on-kinetics equals 7.1, 8.2, 9.4, 10.5, 11.7, 12.8 and 13.9 mM·min^−1^ for A_UT_ = 60, 70, 80, 90, 100, 110 and 120, respectively. *t*_0.63_ changes little with work intensity (see [17] for discussion) and slightly increases from 24.2 s at A_UT_ = 60 to 25.6 s at A_UT_ = 110.

For A_UT_ = 60, muscle V̇O_2_ stabilizes at a steady-state soon after (2–3 min) the onset of exercise and the actual V̇O_2_ on-kinetics overlaps with the primary phase II of the V̇O_2_ on-kinetics. This is the M domain.

For A_UT_ = 70, the muscle V̇O_2_ on-kinetics first follows the exponential primary phase II kinetics, and then, after less than 2 min of exercise, the slow component of the muscle V̇O_2_ on-kinetics is activated due to P_i_ reaching Pi_crit_; this transition generates a characteristic ‘notch’ in the V̇O_2_ on-kinetics, at least for some ATP usage activities. However, afterwards, V̇O_2_ ultimately stabilizes at a greater V̇O_2_ than expected based on phase II V̇O_2_ kinetics, but below V̇O_2max_. A significant slow component can be observed. This simulation represents the H domain.

For A_UT_ = 80, 90, 100 and 110, muscle V̇O_2_ reaches V̇O_2max_ and the higher the A_UT_, the sooner exercise is terminated. Here, the absolute magnitude of the slow component decreases with A_UT_. The primary phase II of the V̇O_2_ on-kinetics does not project above V̇O_2max_. This is the VH domain.

Finally, for A_UT_ = 120, V̇O_2_ rapidly attains V̇O_2max_, at which exercise is terminated, the slow component is very small as it has very little time to develop and the actual V̇O_2_ on-kinetics is difficult to discern from the primary phase II of the V̇O_2_ on-kinetics. The primary phase II of the V̇O_2_ on-kinetics projects above V̇O_2max_. Therefore, this simulation represents S domain.

**Figure 3:**
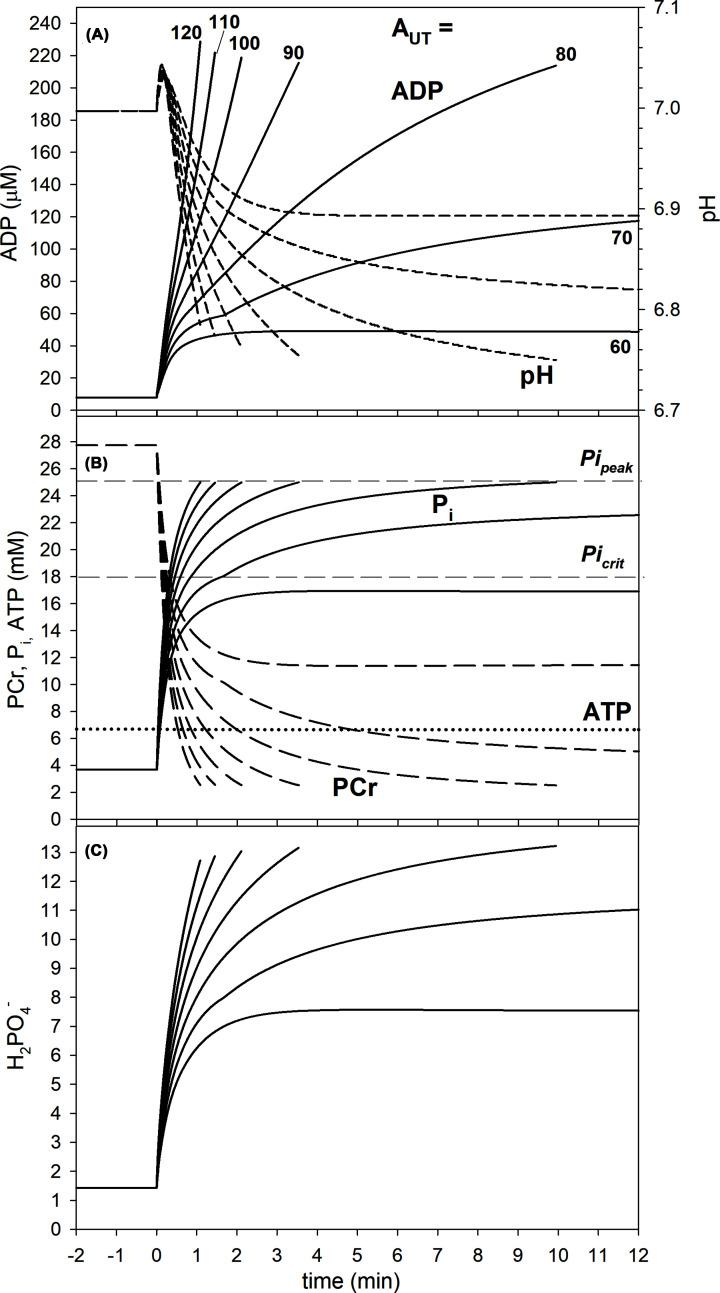
Simulated on-kinetics of selected metabolites of the skeletal muscle bioenergetic system (**A**) ADP and pH; (**B**) PCr, P_i_ and ATP; (**C**) H_2_PO4^−^.

Muscle phosphate metabolite concentrations (ADP, PCr, Pi, H_2_PO_4_^−^) and pH follow a similar pattern. This is shown in Figure 3. Metabolites also quickly reach a steady-state for A_UT_ = 60, reach a delayed and elevated steady state, with a ‘notch’ in their kinetics, for A_UT_ = 70 and absolute concentration changes are more rapid with increasing A_UT_ values. For A_UT_ values = 80 and above, end-exercise metabolite concentrations are identical for PCr and for P_i_ (in the latter case by definition) and similar for ADP, H^+^ and H_2_PO4^−^. The relative increase in H_2_PO4^−^ is larger (9.1 times for A_UT_ = 100) than in P_i_ (6.8 times), as the former is a derivative of both P_i_ and H^+^ increase.

**Figure 4:**
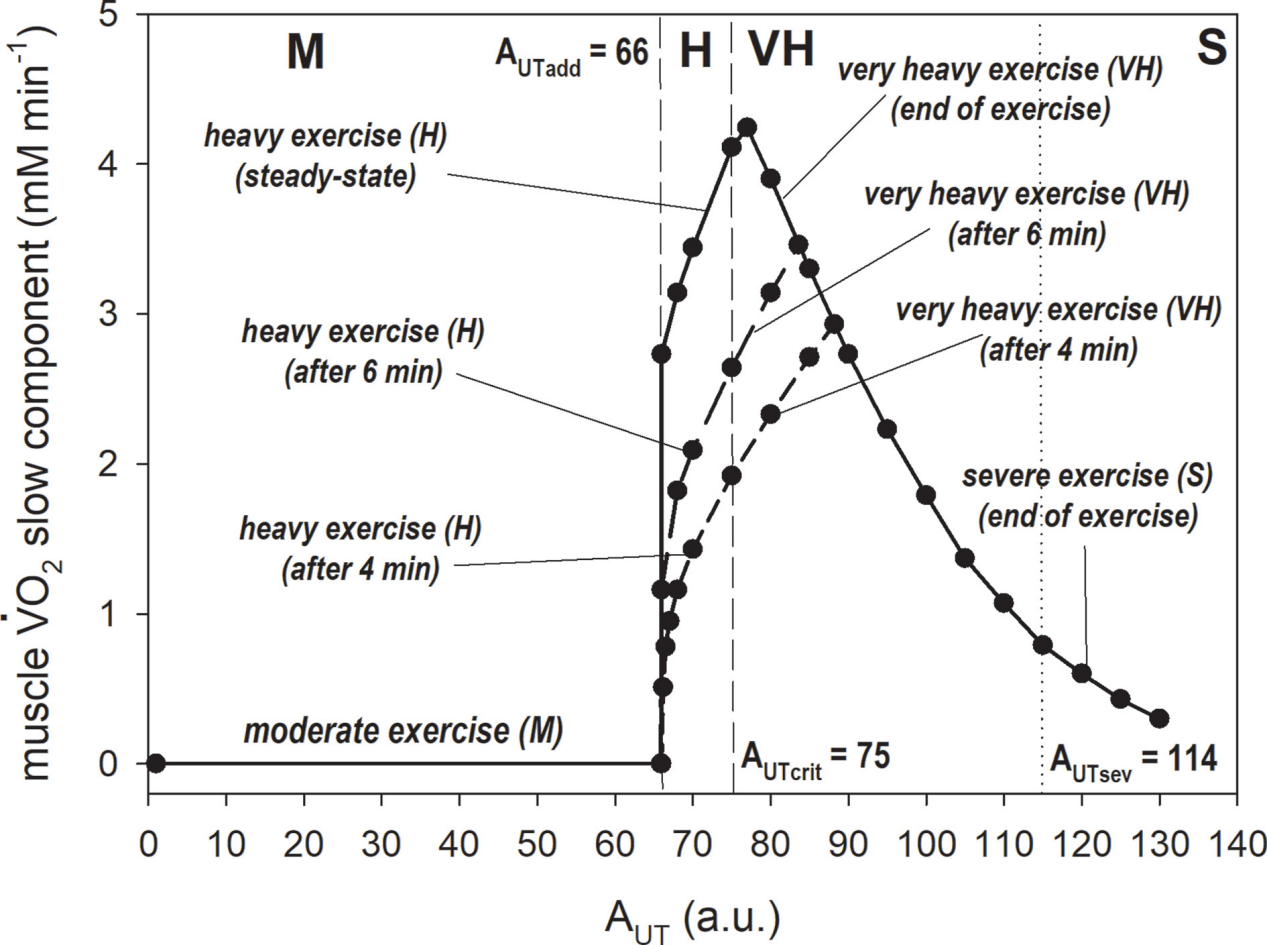
Simulated dependence of the muscle V̇O_2_ slow component on ATP usage activity (A_UT_, corresponding to PO) Slow component values at the termination of exercise are shown (upper curve; solid line). In addition, slow component values after 4 and 6 min of exercise are shown (lower two curves; dash line). Exercise intensity domains are separated by vertical dashed or dotted lines. The values of ATP usage activity (A_UT_) at M/H border (A_UTadd_), H/VH border (A_UTcrit_) and VH/S border (A_UTsev_) are indicated.

The absolute value of the V̇O_2_ on-kinetics slow component, by definition absent in M domain, increases with A_UT_ to maximal value immediately above the H/VH border. Then it decreases with A_UT_ from the maximal value to low values in VH domain, and from low to very low values in the S domain. This is presented in Figure 4. In the S domain, the slow component has simply too little time to fully develop before termination of exercise because of fatigue.

The simulated values of A_UT_ at the M/H border (A_UTadd_), H/VH border (A_UTcrit_) and VH/S border (A_UTsev_) for normal, physically active muscle are presented in Figure 4 and in the middle row of Table 1. They equal A_UT_ = 66, 75 and 114, respectively. The difference between A_UTadd_ and A_UTcrit_ (here, termed the ‘H space’) equals 9, while the difference between A_UTcrit_ and A_UTsev_ (or ‘VH space’) equals 39. In sedentary individuals (untrained muscle), the values of A_UTadd_, A_UTcrit_ and A_UTsev_ are lower, than in normal muscle, H space is greater, while VH space is approximately the same (top row in Table 1). In endurance-trained muscle, A_UTadd_, A_UTcrit_ and A_UTsev_ are greater than in normal physically active muscle, H space is reduced, while VH space is approximately the same (bottom row in Table 1). Thus, the training status is one of the factors determining the values of the M/H, H/VH and VH/S borders and the space between them.

**Table 1:** ATP usage activities at the borders between exercise intensity domains

Training status	ATP usage activity (a.u.)				
	A_UTadd_	A_UTcrit_	A_UTsev_	H space	VH space
Untrained *k*_OX_ × 0.8, Pi_peak_ = 27 mM	52	68	107	16	39
Physically active * k*_OX_ × 1.0, Pi_peak_ = 25 mM	66	75	114	9	39
Endurance trained *k*_OX_ × 1.1, Pi_peak_ = 24 mM	73	79	116	6	37

The values of ATP usage activity at the border between the moderate (M) and heavy (H) exercise domain (A_UTadd_), between the heavy and very heavy (VH) exercise domain (A_UTcrit_) and between the very heavy and severe (S) exercise domain (A_UTsev_). The space for heavy exercise: H space = A_UTcrit_ - A_UTadd_. The space for very heavy exercise: VH space = A_UTsev_ - A_UTcrit_.

## Discussion

### Biochemical origins of muscle exercise intensity domains

This study aimed to determine the origin of skeletal muscle exercise intensity domains at the biochemical/molecular level. In particular, our aim was to delineate these domains in terms of the ‘P_i_ double-threshold’ mechanism of muscle fatigue, comprising the P_i_ on-kinetics, Pi_crit_, Pi_peak_ and the kinetics of the dependence of the additional ATP usage on the P_i_-Pi_crit_ difference.

We postulate that, in the M domain, the ATP usage activity (A_UT_) is too small (below A_UTadd_) for P_i_ to exceed Pi_crit_. Therefore, the additional ATP usage is not initiated and the system (fluxes and metabolite concentrations) quickly reaches a steady state. In the H domain, A_UT_ is high enough (larger than A_UTadd_) to cause P_i_ to exceed Pi_crit_ but too low (smaller than A_UTcrit_) to bring P_i_ (at a given additional ATP usage kinetics) to Pi_peak_. Therefore, the additional ATP usage increases only temporarily, the slow component appears for a time, but ultimately the system stabilizes, albeit at a higher V̇O_2_ than that expected from the primary phase II kinetics (i.e., the additional ATP usage-P_i_ positive feedback loop is too weak to cause a continuous increase in the additional ATP usage). In the H domain, V̇O_2_ does not reach V̇O_2max_ and exercise is not terminated because of fatigue (at least for the 30-min duration simulated here).

The value of A_UT_ that is great enough to cause P_i_ and additional ATP usage to progressively increase throughout exercise, where P_i_ eventually reaches Pi_peak_, is termed A_UTcrit_. A_UTcrit_ is an emerging feature of the bioenergetics system, and not a pre-determined value of A_UT_ or P_i_ (or other metabolite(s)). Below A_UTcrit_ the positive feedback signal posed by mutual stimulation of P_i_ increase and additional ATP usage increase is not strong enough for P_i_ to reach Pi_peak_ (and for V̇O_2_ to reach V̇O_2max_) and V̇O_2_ and P_i_ and other metabolites can eventually stabilize. A_UTcrit_ therefore is determined by the work rate (reflected in the absolute ATP usage activity) and the properties of the system itself, e.g. OXPHOS activity, ESA activity, Pi_peak_, Pi_crit_, k_add_ (activity of the additional ATP usage) or O_2_ concentration etc. [12,13]. When A_UT_ exceeds A_UTcrit_, the mutual stimulation (positive feedback) of P_i_ increase and additional ATP usage increase is strong enough for P_i_ to increase progressively throughout exercise and ultimately reach Pi_peak_. At the same moment, V̇O_2_ reaches V̇O_2max_ and exercise is terminated because of fatigue. If A_UT_ < A_UTsev_, the primary phase II of the V̇O_2_ on-kinetics does not project above V̇O_2max_ and the muscle is within the VH domain. If A_UT_ > A_UTsev_, the primary phase II of the V̇O_2_ on-kinetics projects above V̇O_2max_ and the muscle enters the S domain.

Regarding the overall V̇O_2_ on-kinetics, there is no sharp border between VH and S intensity domains, as the VH domain passes smoothly (continuously) into the S domain. Rossiter [1] argued that in S intensity domain the slow component cannot be discerned from the primary phase II, rather than that it does not appear at all. The ‘P_i_ double-threshold’ approach supports this point of view, as the additional ATP usage, and thus the slow component, is always initiated once P_i_ exceeds Pi_crit_.

Thus, using our model, the muscle exercise domains may be characterized at the biochemical/molecular level in skeletal muscle fibers as follows and are detailed in Table 2:

- M domain – P_i_ does not reach Pi_crit_; no slow component is present; a steady-state is quickly reached; V̇O_2_ does not reach V̇O_2max_
- H domain – P_i_ exceeds Pi_crit_ but does not reach Pi_peak_; a slow component is present, but a delayed steady-state is reached; V̇O_2_ does not reach V̇O_2max_
- VH domain – P_i_ exceeds Pi_crit_ and ultimately reaches Pi_peak_; V̇O_2_ reaches V̇O_2max_; exercise is terminated because of fatigue; the primary phase II of the V̇O_2_ on-kinetics does not exceed V̇O_2max_; the slow component of the V̇O_2_ (and metabolites) on-kinetics is required to bring V̇O_2_ to V̇O_2max_
- S domain – P_i_ exceeds Pi_crit_ and ultimately reaches Pi_peak_; V̇O_2_ reaches V̇O_2max_; exercise is terminated because of fatigue; the slow component of the V̇O_2_ (and metabolites) on-kinetics has little time to develop, because the primary phase of the V̇O_2_ on-kinetics exceeds V̇O_2max_

**Table 2:** Exercise intensity domains defined within the ‘P_i_ double-threshold’ mechanism of muscle fatigue

Property	Intensity domain			
	Moderate (M)	Heavy (H)	Very heavy (VH)	Severe (S)
Pi_crit_ Exceeded	No	Yes	Yes	Yes
Pi_peak_ Reached	No	No	Yes	Yes
Steady-state	Yes	Yes	No	No
Positive Feedback	No	Moderate	High	Very high
Slow Component	No	Moderate → High	High → Low	Low → Very low
Phase II V̇O_2_ exceeds V̇O_2max_	No	No	No	Yes

Particular domains are characterized by selected system properties.

It should be emphasized that the ‘P_i_ double-threshold’ mechanism used in this model is a deliberate simplification of the complex and numerous processes leading to muscle fatigue, reduced work efficiency and contributing to exercise intolerance. This is discussed in more detail in the "Study limitations" section.

It should also be clearly emphasized that Pi_crit_ is directly related to A_UTadd_, and not A_UTcrit_ (analogous to CP). Pi_crit_ is a parameter, while A_UTcrit_ is an emergent property of the system, especially of the dependence of the additional ATP usage intensity on the P_i_-Pi_crit_ difference (involving the ‘rate constant’ of the additional ATP usage, *k*_add_), Pi_crit_ value and Pi_peak_ value (see [12,13]). A_UTsev_ is clearly related to Pi_peak_ (affecting V̇O_2max_). A_UTadd_, A_UTcrit_ and A_UTsev_ are also co-determined by the OXPHOS activity and ESA intensity, as they affect changes in P_i_ during exercise [12,13].

### Muscle exercise intensity domains versus whole-body V̇O_2_ and blood L^−^ kinetics

Of course, it is expected that exercise intensity domains at the muscle level underlie those at the whole-body level. However, a question arises whether the borders between the domains at both levels strictly overlap.

Using this model, the muscle and whole-body intensity domains can be related to each other through a conversion factor: one A_UT_ unit of muscle ATP usage intensity is equivalent to about 3 W (2–4 W depending e.g. on working muscles mass) of the whole-body power output during cycling. This allows a relative scaling to be established between e.g. muscle A_UTcrit_ and whole body CP, or % of muscle maximal A_UT_ and whole-body PO_max_ in ramp-incremental exercise. Alternatively, A_UT_ can be described by muscle PO per unit muscle mass expressed in watt/kg.

It is not obvious that the skeletal muscle exercise intensity domains, determined mostly at the biochemical and molecular level, and whole-body exercise intensity domains at the physiological level, determined mostly on the basis of the pulmonary V̇O_2_ on-kinetics and blood L^−^ (and CO_2_) on-kinetics, should precisely overlap in each case. For instance, the whole-body M/H border determined by the V̇O_2_ on-kinetics and LT/GET could potentially differ from the muscle M/H border, which depends on P_i_ exceeding Pi_crit_. In particular, there seems to be no necessary reason that the fraction of the pulmonary slow component, originating predominantly in working muscles and in other tissues, should appear at the same time and PO/A_UT_. The M/H border is defined at the whole body level by the emergence of the pulmonary V̇O_2_ slow component and/or the failure of blood L^−^ to stabilize at (or close to) resting values and is analogous to LT/GET. On the other hand, M/H boarder in the muscle is defined by the highest A_UT_ that does not cause P_i_ to exceed Pi_crit_. LT cannot be defined at the molecular/cellular level (single muscle fiber level) in the same way as it is at the whole-body (blood) level because blood lactate concentration during exercise is a result of the balance between lactate release by working muscle fibers and lactate uptake by non-working fibers and other tissues. Cytosolic lactate is a derivate of the rate of lactate/H^+^ production by anaerobic glycolysis and the rate of lactate/H^+^ efflux to blood. Consequently, some cytosolic acidification (noting also that muscle buffering capacity is less than the blood), and likely elevated cytosolic lactate concentration, is already present at POs/A_UT_s that would be considered M exercise as defined at the muscle level by P_i_ exceeding Pi_crit_, or at the whole body level by pulmonary V̇O_2_ on-kinetics or blood L^−^ measurements (see e.g., Cannon et al., 2014, where some muscle acidification appears in what is otherwise considered M exercise).

Poole et al. [18] showed that the contribution of working muscles to the whole body V̇O_2_ slow component is ∼80% in VH exercise achieving V̇O_2max_ in approximately 20 min; other tissues such as cardiac, respiratory and accessory/stabilizing muscles are presumed to contribute the remaining ∼20%. However, at lower POs in the H and VH domains, the fractional contribution of other tissues to the whole body V̇O_2_ slow component may be greater. Recognizing that other tissues contribute to the V̇O_2_ slow component, supports the idea that it is not necessary for the M/H border to occur at an identical PO/A_UT_ at the whole body and muscle levels. Early muscle acidification and lactate accumulation, coupled with the contribution of other tissues to the pulmonary V̇O_2_ slow component, suggest that it is possible for the whole body M/H boarder to occur at a lower PO, and the V̇O_2_ slow component to start earlier in time, than their equivalents in the working muscle. Nevertheless, according to the present knowledge this is speculation. On the other hand, some dissociation of the pulmonary and muscle V̇O_2_ slow component kinetics can be seen in [19] (Figure 9A therein).

It is also possible that the M/H border determined from the pulmonary V̇O_2_ on-kinetics (lack or presence of the slow component) has a somewhat different value (in Watts or Watts per working muscle mass, for instance) than the M/H border determined from L- and H^+^ increase in blood. There seems to be no causal relation between the onset of the (pulmonary) V̇O_2_ slow component, constituting, in fact, an ‘excessive’ oxygen uptake at a given work intensity and elevated lactate concentration in blood (despite the strong corellative association between these variables) [20]. In fact, an increase in the rate of anaerobic glycolytic ATP supply alone (that produces lactate), provided that all other variables are kept unchanged, would decrease oxidative ATP supply, and thus V̇O_2_ (the Crabtree effect), and not cause a disproportional increase (slow component). Also the elevated oxygen consumption by ‘other tissues’ is unlikely to be directly associated with elevated blood L^−^, blood acidification or increased partial pressure of CO_2_, becuase these tissues preferentially consume L^−^ as a respiratory substrate. Finally, the L^−^ concentration in arterial blood is a derivative of the balance between the L^−^ release by active muscle and its uptake by other muscle and other tissues [21]. This balance does not have to be directly causally linked with the V̇O_2_ on-kinetics in active muscle or other tissues. Therefore, the similar values of the M/H border determined on the basis of the V̇O_2_ on-kinetics and from LT/GET seem likely to be an indirect association, related, but not directly causally linked with the event of P_i_ exceeding Pi_crit_.

On the other hand, in our opinion, the H/VH border is well- and uniquely-defined in terms of CP/A_UTcrit_, both at the biochemical muscle and physiological whole-body level. It can be characterized as the highest PO/A_UT_ at which P_i_ and V̇O_2_ is able to stabilize and therefore P_i_ does not reach Pi_peak_ and V̇O_2_ does not reach V̇O_2max_. This behavior is a result of the intrinsic bioenergetic properties of the muscle. While the point of initiation (in time, PO or working muscle mass) of the V̇O_2_ and metabolite slow component can differ between the whole-body and biochemical muscle levels, it is the muscle bioergetic properties that determine muscle fatigue and the termination of exercise related to it (when P_i_ reaches Pi_peak_), both directly and through the action of the feedback to central nervous system [22–25].

In light of the ongoing debate about how to best characterize the highest PO at which whole-body physiologic variables stabilize, i.e. the H/VH border e.g. [26,27], it is worth noting that the H/VH borders determined on the basis of CP, maximal lactate steady-state (MLSS) or respiratory compensation point (RCP) do not have to overlap. The reason is that CP is a property of, and generated within, active muscles (see [14] for discussion), while MLSS and RCP are systemic events not only related to the muscle-generated CP but also influenced by other factors (the balance of lactate appearance and clearance, the senstitvity of the carotid body to an acidosis, or the absence of mechanical constraints limiting ventilation; [21,28,29]). We prefer to define the H/VH border on the basis of the active muscle-generated CP, as its intramuscular origin is unequivocal, is directly associated with muscle fatigue, and preceeds the later occruing systemic physiologic events.

The VH/S border can be defined, somewhat more abstractly, as PO/A_UT_ at which P_i_ would stabilize at (just below) the Pi_peak_ value, and V̇O_2_ would stabilize just below V̇O_2max_, were the additional ATP usage, and thus the slow component of the V̇O_2_ on-kinetics, to be absent (‘switched off’).

### The magnitude of the slow component across exercise intensity domains

A dependence of the absolute value of the pulmonary V̇O_2_ slow component on PO was extracted in Poole and Jones [2] from experimental data presented in Poole et al. [30]. The simulated dependence of the muscle V̇O_2_ slow component on A_UT_, shown here in Figure 4, is quite similar. The main difference between the computation and experimental data is that the simulation results in a narrower H space and a faster increase in slow component magnitude just above the M/H border. However, the experimental data contain only one point for the H domain, concealing a more detailed comparison.

At the muscle level in Figure 4, the H/VH border represents ∼63–68% of the A_UTsev_, which corresponds well to values of CP from whole body exercise that average 70% of the PO at V̇O_2_max (range: 53–80%, [5,31]). However, Figure 4 shows that the H/VH border is relatively low in the H+VH space range; it is ∼15–30% of the A_UT_ range between the M/H boarder and the VH/S border (depending on the training status of the muscle). This range, termed the ‘delta’ (%Δ) range in whole body studies e.g., Whipp (1996), is lower than expected based on CP from whole body exercise, which varies between ∼15 and 60%Δ in healthy subjects [5,31]. This again supports the notion that the M/H boarder in whole body exercise may occur at a lower PO/A_UT_ than its muscle equivalent, allowing the %Δ at which CP occurs to be greater at whole body compared with the muscle level. In addition, the VH/S boarder may also be lower in whole body exercise than at the muscular level, particularly in the trained state where muscle OXPHOS capacity (at least) can exceed the capacity for whole body O_2_ delivery [32,33]. Therefore, both the M/H and VH/S borders may be significantly lower in whole body exercise, and thus the H space significantly broader in whole-body exercise compared with the isolated muscle.

### V̇O_2_ on-kinetics generation in particular exercise intensity domains

**Figure 5:**
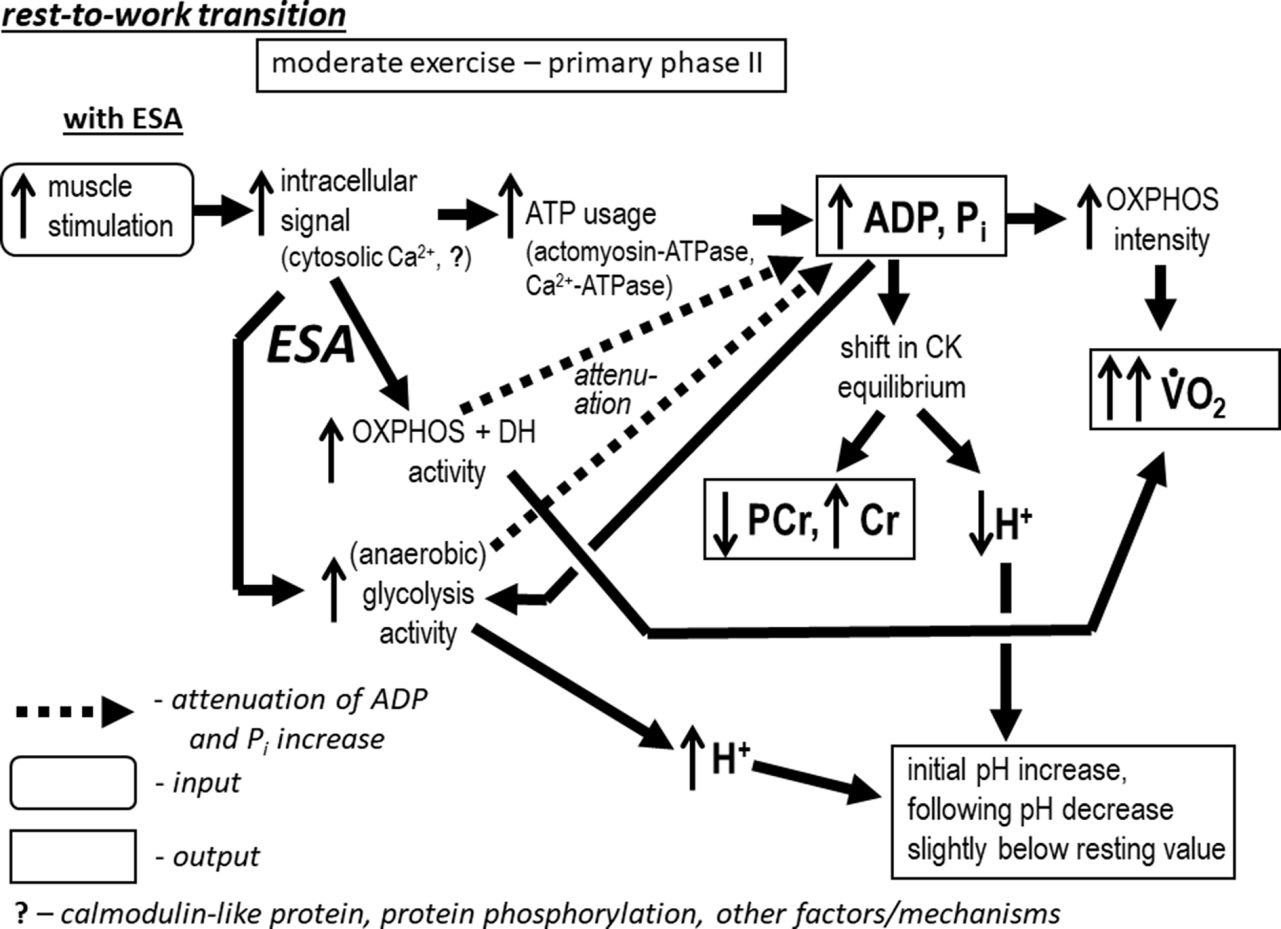
Biochemical background of the primary phase II of the V̇O_2_ and metabolites on-kinetics Sequence of biochemical/molecular events (causal chain) in the bioenergetic system during rest-to-work transitions in skeletal muscle below the M/H border (M exercise intensity domain) in the presence of ESA. A detailed description is provided in the text.

**Figure 6:**
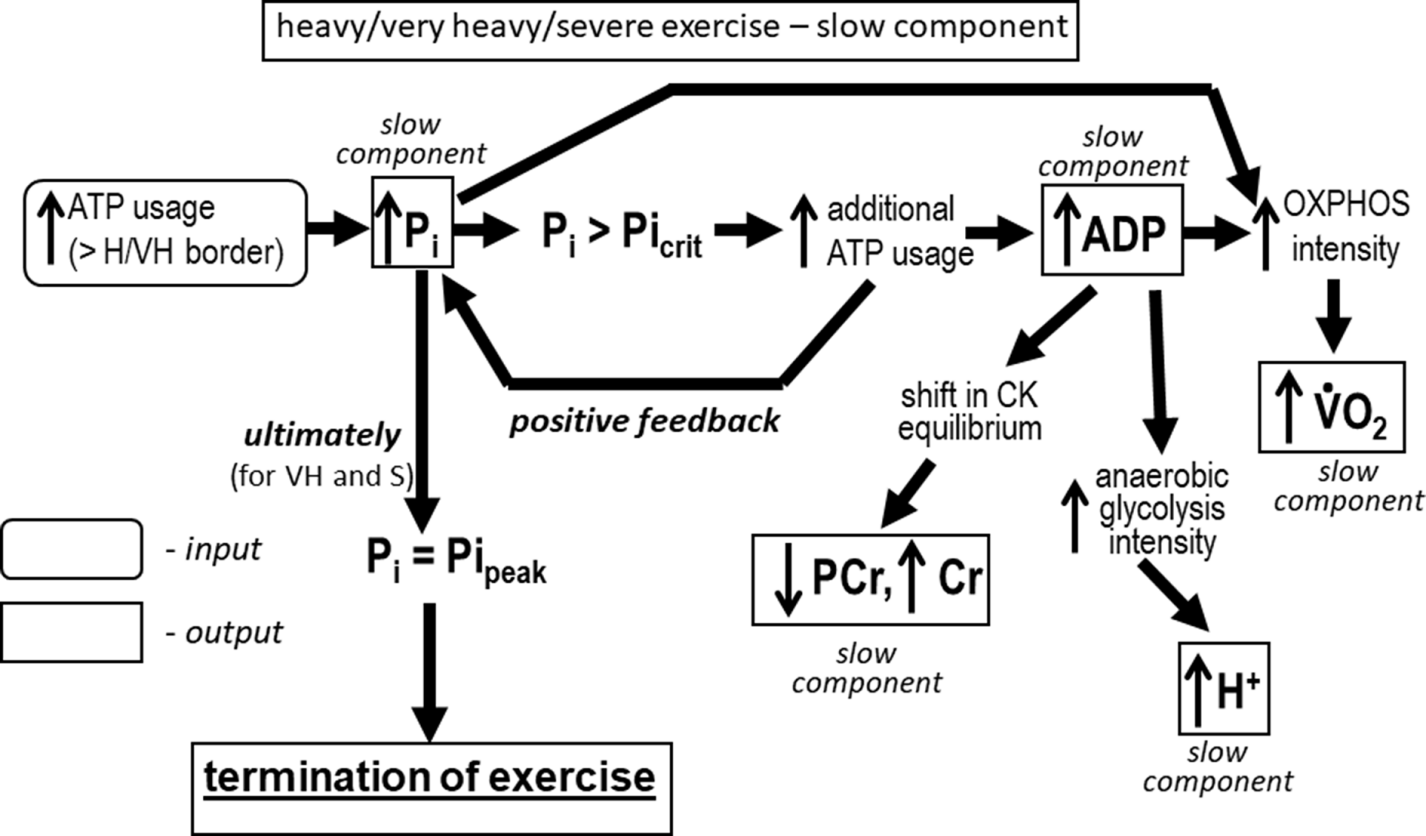
Biochemical background of the slow component of the V̇O_2_ and metabolites on-kinetics Sequence of biochemical/molecular events (causal chain) in the bioenergetic system during rest-to-work transitions in skeletal muscle during exercise above the M/H boarder (H, VH and S exercise intensity domains) in addition to the system behavior below the M/H border depicted in Figure 5 (primary phase II of the V̇O_2_ and metabolites on-kinetics). A detailed description is provided in the text.

The V̇O_2_ on-kinetics in particular exercise intensity domains is an emergent property (epiphenomenon) of the biochemical bioenergetic system of skeletal muscle. Figures 5 and 6 describe the causal chain (sequence of events) from the input (muscle stimulation) to the outputs (chiefly V̇O_2_) in the primary phase II (Figure 5) and slow component (Figure 6) of the system on-kinetics. In this chain, preceding factors (before arrows: e.g. enzyme/metabolic block activities and metabolites) influence following factors (after arrows: enzyme/metabolic blocks activities, fluxes and metabolite concentrations) but not inversely. V̇O_2_ is a consequence of this chain of events and as such is not a causal factor of system function; rather it is an epiphenomenon that may be used to non-invasively identify biochemical events originating in the muscle.

The sequence of biochemical/molecular events (causal chain) in the bioenergetic system during rest-to-work transition in skeletal muscle during moderate (M) exercise (primary phase II of the V̇O_2_ and metabolites on-kinetics) in the presence of each-step activation (ESA) is presented in Figure 5. Neural myocyte stimulation causes a release of Ca^2+^ ions from sarcoplasmic reticulum. This in turn activates actomyosin-ATPase (muscle contraction) and Ca^2+^-ATPase (SERCA). ATP is hydrolyzed to ADP and P_i_, which elevates the concentration of the two latter (ATP concentration remains approximately constant because of the high ATP/ADP ratio, unless AMP deamination leads to a decrease of the total pool of adenine nucleotides). In parallel, essentially all elements of the system (perhaps with exception of the very fast CK), both cytosolic and mitochondrial, are directly activated by some factor/mechanism, probably related to Ca^2+^, but likely involving also some other elements, e.g., calmodulin-like protein(s), ‘presenting’ Ca^2+^ ions to enzymes/carriers and/or protein phosphorylation. This attenuates the increase in ADP and P_i_ (in relation to the situation without ESA, [34], as lower accumulation of these metabolites is necessary in order for oxidative (and glycolytic) ATP supply to match the elevated ATP usage, because OXPHOS (and glycolysis) is already partly activated by ESA. The moderate ADP increase shifts the equilibrium of creatine kinase (CK), which leads to a moderate PCr decrease, Cr increase, consumption of H^+^ (initial pH increase) and further moderate P_i_ increase (concomitant action of CK and ATP usage). The moderate increase in ADP and P_i_ stimulates OXPHOS, which leads to a significant increase in V̇O_2_ (OXPHOS is activated directly, and in parallel, through ESA). Because of moderate changes in metabolite concentrations, especially PCr, Cr and P_i_, the transition time of the primary phase II of the V̇O_2_ and metabolites on kinetics (*t*_0.63_ or τ_p_) is relatively short. ADP (and AMP) increase further activates (anaerobic) glycolysis. Production of H^+^ by anaerobic glycolysis can slightly decrease pH below the resting value (e.g. [35]). However, accumulating protons inhibit (anaerobic) glycolysis, which prevents further significant cytosol acidification. Ultimately, the system reaches a steady-state.

Exercise in the H, VH and S domains entail an additional sequence of biochemical/molecular events (causal chain) in the muscle bioenergetic system supplementing the primary phase II of the system on-kinetics. This sequence of events underlies the slow components of the V̇O_2_ and metabolite on-kinetics (Figure 6). A work intensity (ATP usage activity) that is sufficiently high to cause P_i_ to exceed critical P_i_ (Pi_crit_) initiates additional ATP usage (above that expected based on phase II kinetics). This, in turn, leads to a further increase in ADP and P_i_, the latter further stimulating additional ATP usage, thereby forming a self-driving process (positive feedback loop). The increased ADP shifts the CK equilibrium, leading to a further decrease in PCr and increase in Cr. ADP (and AMP) further stimulates anaerobic glycolysis, which causes greater cytosol acidification. This in turn recursively inhibits (anaerobic) glycolysis (self-limiting process). The continuously increasing ADP and P_i_ stimulate OXPHOS and thus lead to a further increase in V̇O_2_. As a result, the slow component in the V̇O_2_, P_i_, PCr, Cr and H^+^ on-kinetics appears. In H exercise, the mutual stimulation of the increase in P_i_ and increase in the additional ATP usage is not strong enough for P_i_ to reach Pi_peak_ and thus for V̇O_2_ to reach V̇O_2max_. As a result, the system ultimately stabilizes, albeit at a higher steady-state than that expected in the absence of the additional ATP usage. The H/VH border is an emerging property of the system that separates POs/A_UT_s for which this feedback loop can stabilize from those for which it cannot, i.e. A_UTcrit_ in the muscle and CP at the whole body level. In VH and S exercise the mutual stimulation of the increase in P_i_ and increase in the additional ATP usage is strong enough to prevent a steady-state from being achieved, V̇O_2_ increases and metabolites change continuously throughout exercise. Ultimately, P_i_ reaches Pi_peak_, V̇O_2_ reaches V̇O_2max_ and the exercise is terminated because of fatigue.

Work performed above CP (W’ parameter of the power-duration dependence) has historically been termed ‘anaerobic work capacity’ or AWC. However, it should be emphasized that, under the conditions presented here for healthy individuals, the vast majority of ATP supply during exercise above CP (A_UTcrit_) is by OXPHOS. Creatine kinase (CK) is the main ATP supplier in the initial seconds of exercise (first 20–30 s) [12,17], but this is also true for power outputs below CP (A_UTcrit_).

Goulding et al. [36] collected numerous whole body experimental data demonstrating a close association between the V̇O_2_ on-kinetics (*t*_0.63_ and/or O_2_ deficit) and various system properties, especially CP. The simulations presented here emphasize that these associations from experimental data are not determined by V̇O_2_ on-kinetics, and that both CP and V̇O_2_ kinetics are emergent properties of the bioenergetic system. In the data presented by Goulding et al. [36], the V̇O_2_ on-kinetics represents a non-invasive characteristic (or proxy) that results from system parameters and variables, such as OXPHOS activity, ESA activity, O_2_ concentration, or Pi_peak_ [13]. The observed association between the V̇O_2_ on-kinetics and CP is consistent with computer simulations in that both these outputs result from parameters and variables of the system [13]. The inverse (negative) correlation between *t*_0.63_ and CP observed in experimental studies results from the fact that the mentioned parameters change *t*_0.63_ and CP in the opposite directions—compare e.g. two upper rows in Table 1 in [13]. A change in, e.g., total phosphate and/or creatine pool would work in a similar way. A similar reasoning can be applied to the V̇O_2sc_ (V̇O_2_ slow component) − W' (curvature constant of the power–duration relationship) relationship. Again, these emergent system properties are determined by several parameters, for instance *k*_add_ - the ‘rate constant’ of the additional ATP usage. It is emphasized that V̇O_2_ and V̇O_2_ kinetics are epiphenomena, located at the end of the causal chains shown in Figures 5 and 6.

### Off-transients versus exercise intensity domains

During muscle recovery (off-transient) P_i_ quickly falls below Pi_crit_ (after ∼10–20 s, see e.g. Figure 5 in [17]). Therefore, according to the ‘P_i_ double-threshold’ mechanism, the additional ATP usage and thus slow component of the muscle V̇O_2_ off-kinetics quickly disappears. This conclusion conforms well to experimental observations concerning recovery after M and H exercise [37]. A slow approach of pulmonary V̇O_2_ to the resting value, resembling to some extent the slow component of the V̇O_2_ on-kinetics, was observed during recovery after VH exercise [37]. However, this phenomenon could be caused, at least partly, by a slow decay of ESA (during recovery OXPHOS produces ATP mostly for PCr resynthesis by CK), slowing the off-transient of the muscle (and therefore also pulmonary) V̇O_2_ off-kinetics [38]. Additionally, Krustrup et al. [19] demonstrated that pulmonary τ_p_ during off-transient is significantly longer, than muscle τ_p_. This can be due to a slow recovery of cardiac and respiratory muscle activity (heart rate and ventilation remain raised for many minutes following VH or S exercise) and/or V̇O_2_ in other tissues, re-filling of O_2_ stores in tissues and blood, and circulatory distortion between muscle and pulmonary V̇O_2_. This conclusion is supported by the fact that such a ‘slow component’ of the V̇O_2_ off-kinetics is observed during recovery from intense exercise on the whole-body (pulmonary) level but not on the muscle level (see Figure 9B in [19]). In this case, the contribution of ESA decaying slowly, is likely low. Generally, the ‘slow component’ of the V̇O_2_ off-kinetics does not seem underlain by the additional ATP usage in working muscles, as is the slow component of the V̇O_2_ on-kinetics.

### General discussion

In constant-power exercise of an isolated muscle group end-exercise PCr, pH and P_i_ are similar for various work intensities [25]. In addition, when exercise tolerance is manipulated using alterations in oxygen delivery, end-exercise P_i_, PCr and pH are similar [23]. These observations support the concept of Pi_peak_. No thresholds are observed in biochemical studies concerning the relationship between P_i_ and force generation in skinned fibers [39,40]. However, this system is very different from voluntary constant-power exercise in intact muscles, as it involves no cytosolic milieu, varying force, constant pH, constant external Ca^2+^, no Ca^2+^ handling and no ATP usage by Ca^2+^-ATPase (SERCA). Unlike skinned fibers, task failure in isolated muscle constant-power exercise occurs when power production is no longer capable of meeting the task requirement, thus occurring at a common magnitude of peripheral fatigue [22,24]. Therefore, skinned fibers and intact muscle systems cannot be directly compared (see [14] for discussion).

The P_i_ double-threshold mechanism can be sensibly defined in some types of exercise including voluntary constant-power exercise and perhaps ramp exercise and all-out exercise, although the values of Pi_peak_ and Pi_crit_ can be different in different exercise types. On the other hand, it is not clear whether this mechanism works in other cases, such as isometric exercise or electrically stimulated muscle.

### Study limitations

The dynamic model used for computer simulations in this study, as any model of this kind, constitutes only a simplification and approximation of the complex reality. For instance, it is a one-compartment model that does not distinguish different muscle fiber types and operates with parameters and variables (activities, fluxes, metabolite concentrations) averaged over the entire muscle. On the other hand, it is compared with ‘one-compartment’ experimental data: muscle (or pulmonary) V̇O_2_ and muscle PCr, P_i_, ADP, ATP and H^+^ concentrations. When doing this, the model is able to account, at least semi-quantitatively, for a surprisingly wide range of various kinetic properties of the skeletal muscle bioenergetic system.

The original ‘P_i_ double-threshold’ mechanism involves explicitly the total concentration of P_i_ as the main peripheral-fatigue-related metabolite. However, it is possible that the deprotonated form of P_i_ – H_2_PO_4_^−^ is the factor that directly leads to muscle fatigue and exercise intolerance [41]. An advantage of this possibility is that the H_2_PO_4_^−^ concentration is a derivative of P_i_ and H^+^ concentrations (acidification increases the fraction of P_i_ being in the form of H_2_PO_4_^−^), considered as two most important fatigue factors [9]. Additionally, the relative increase of H_2_PO_4_^−^ during rest-to-work transition is greater than that of P_i_ (9.1-fold versus 6.8-fold increase, see Figure 3). When P_i_ is substituted by H_2_PO_4_^−^ within the computer model, similar general theoretical results are obtained, although, of course, with different critical and peak values of H_2_PO_4_^−^ (not shown).

The ‘P_i_ double-threshold’ mechanism is a deliberate simplification of the complex and numerous processes leading to muscle fatigue, reduced work efficiency and contributing to exercise intolerance. The precise quantitative details of these processes are yet to be determined, but most probably include action of other variables, especially H^+^ and alteration in Ca^2+^ release and sensitivity. On the other hand, as it is discussed in Korzeniewski [14], P_i_ can cause Ca^2+^ precipitation in sarcoplasmic reticulum and mediate in central fatigue (the central nervous system can sense somehow the metabolic state of working myocytes). For this reason, P_i_ can be involved, directly or indirectly, also in Ca^2+^-related and central fatigue, which might underlie the excellent agreement between computer simulations of the muscle cell and experimental data from intact working humans. Therefore, the P_i_ double-threshold mechanism provides a useful working hypothesis, which produces quantitative features consistent with physiologic observation.

In addition, the extent to which whole-body V̇O_2max_ (as traditionally defined) is affected by systemic processes (such as convective and/or diffusive O_2_ transport), rather than intramuscular limitations (potentially mediated by P_i_, or neural feedback modulating motor activity), is dependent on the state of training [10,32]. In the skeletal muscle model used here, muscle V̇O_2max_ is effectively affected by Pi_peak_, OXPHOS activity, ESA intensity and O_2_, and the resultant behavior is consistent empirically with observations of V̇O_2max_ during whole-body exercise [12–14]. Nevertheless, increased limitations in O_2_ supply and/or motor activation, or other non-muscle-molecular mechanisms, would be expected to reduce A_UTsev_, lower the VH/S boarder and V̇O_2max_, and reduce the VH space, compared with the simulations presented here. It was shown [12] that a decrease in O_2_ concentration decreases CP/A_UTcrit_, and thus diminishes the H/VH border and H space.

Of course, at the present stage the P_i_ double-threshold mechanism of muscle fatigue is only a hypothesis. Nevertheless, it can account for a surprisingly broad range of various, apparently unrelated, system properties [12–16]. In addition, recent experimental evidence appears broadly consistent with the Pi_crit_ and Pi_peak_ concepts [42]. Therefore, while this mechanism constitutes at best only a simplification and approximation of the reality, it contains properties that closely relate to experimental observations and therefore seem likely to contain at least some construct validity. Certainly, it will have to be ultimately verified or falsified by additional experimental studies. On the other hand, this concept has already stimulated and directed further experimental investigations [42]. The detailed molecular mechanism of the additional ATP usage underlying the slow component of V̇O_2_ and metabolites is not known (some possibilities are discussed in [14]) and will also have to be revealed in the experimental way. Nevertheless, the P_i_ double-threshold mechanism can be regarded as a step towards a more detailed understanding of the phenomenon of muscle fatigue during constant power exercise.

Undoubtedly, the present model is still, to a significant extent, phenomenological, as it does not involve, e.g., the molecular mechanism driving additional ATP usage. Therefore, more detailed models will have to be developed constituting a refinement or extension of the present model.

## Conclusions

A detailed biochemical mechanism, through which the exercise intensity domains in skeletal muscle, namely moderate (M), heavy (H), very heavy (VH) and severe (S) intensity domains, originate at the biochemical/metabolic level of the myocyte is postulated. The genesis of exercise intensity domains and biochemical events in the skeletal muscle myocyte at the onset of exercise involves ESA regulation mechanism and is based on the ‘P_i_ double-threshold’ mechanism of muscle fatigue of a well-tested dynamic computer model of the skeletal muscle bioenergetic system developed previously. The ‘P_i_ double-threshold’ mechanism of muscle fatigue, a necessary simplification of complex system behaviors, is able to generate many various, apparently unrelated, system properties in sedentary, physically-active and endurance-trained muscle that reveal the muscular origins of the M/H, H/VH and VH/S exercise intensity boarders. Muscle training elevates the work intensities at which the M/H and H/VH borders appear and reduces the ‘H space’ i.e. the distance between these borders. It is argued that the value of PO (per working muscle mass) at the M/H border may be different (typically greater) at the skeletal muscle biochemical level compared with the whole-body physiological level. On the other hand, the PO at the H/VH border, above which a steady-state cannot be reached, seems identical in working skeletal muscle and whole-body exercise, and originates mostly in the former. Overall, this study demonstrates how characteristic physiologic responses to exercise over a wide range of intensities emerge, at least in part, from biochemical events at the level of the working skeletal muscle.

## Theoretical methods

### Computer model

The previously developed computer model of the skeletal muscle bioenergetic system, including detailed kinetic OXPHOS description, was used [12,17,43–46]. The model involves the ESA (parallel activation) mechanism, according to which ATP usage, NADH supply, glycolysis/glycogenolysis and all OXPHOS complexes are directly activated by some cytosolic factor/mechanism (likely to involve cytosolic Ca^2+^ ions) during rest-to-work or low-to-high-work transitions in skeletal muscle, heart and other tissues [47,48]. A similar idea was proposed by Fell and Thomas in relation to other metabolic pathways, especially glycolysis [49,50]. The complete model description is given in [14] and located on the website: http://bernardkorzeniewski.pl.

A scheme of the skeletal muscle bioenergetic system is shown in Figure 1. The components of the system that are explicitly considered within the model are presented. The model comprises two main parts. The first is the set of kinetic equations that describe the dependence of the rate of particular enzymatic reactions, processes and metabolic blocks (NADH supply, glycolysis, ATP usage) on metabolite (substrate and product) concentrations. The second is the set of ordinary differential equations that describe the rates of change of particular metabolite concentrations in time: they equal the difference between the rates of all reactions/processes producing a given metabolite and the rates of all reactions/processes consuming it. These two parts form a recurrent, recursive loop: in each simulation time step new reaction/process rates are calculated on the basis of current metabolite concentrations, and new metabolite concentrations are calculated on the basis of current reaction/process rates.

This model was widely tested and was demonstrated to be able to reproduce a broad range of apparently unrelated kinetic properties of the skeletal muscle bioenergetic system, and was used for numerous theoretical studies [12–15,48].

### Computer simulations

Rate constants that appear in kinetic equations for all OXPHOS complexes (complex I, complex III, complex IV, ATP synthase, ATP/ADP carrier, P_i_ carrier) and NADH supply block within the computer model (*k*_C1_, *k*_C3_, *k*_C4_, *k*_SN_, *k*_EX_, *k*_PI_, *k*_DH_, respectively) can be grouped into a single rate constant of OXPHOS: *k*_OX_, which corresponds to OXPHOS activity. In the standard model version, corresponding to normal, physically active individuals, the relative *k*_OX_ is scaled to 1.

In this study three training states are considered, with three different values of *k*_OX_ and Pi_peak_ (compare [12,13]):

1. Normal, physically active individuals, *k*_OX_ = 1.0, Pi_peak_ = 25 mM
2. Untrained, sedentary individuals, *k*_OX_ = 0.8, Pi_peak_ = 27 mM
3. Endurance-trained individuals, *k*_OX_ = 1.1, Pi_peak_ = 24 mM

The time course of selected variable values (total muscle V̇O_2_, muscle V̇O_2_ of the primary phase II, cytosolic ADP, pH, ATP, PCr, P_i_ and H_2_PO4^−^) during rest-to-work transition for increasing ATP usage activity (proportional to PO) (A_UT_ = 60, 70, 80, 90, 100, 110, 120, scaled to 1 at rest) were simulated. It should be noted that total A_UT_ comprises resting A_UT_ = 1 (for basic processes sustaining the functioning of the cell) and unloaded-work-related A_UT_ = 4. The additional ATP usage (giving rise to the V̇O_2_ and metabolite slow components) is a function of the current P_i_–Pi_crit_ difference, and is initiated when P_i_ exceeds the critical value (Pi_crit_ = 18 mM). Simulated exercise termination was defined as when P_i_ reaches Pi_peak_ [12]. Simulations comprised 30 min of exercise unless exercise was terminated sooner because of fatigue.

One A_UT_ unit corresponds to approximately 3 W during whole body exercise (e.g. cycling). This value may vary (between about 2 and 4 W), depending on e.g. working muscle mass and type of exercise. Particular OXPHOS complexes, NADH supply block and glycolysis were activated with some delay in parallel with ATP usage at the onset of exercise through ESA (see e.g. [45]).

The values of the ATP usage activity (A_UT_, analogous to PO) corresponding to the M/H border, H/VH border and VH/S border have been named A_UTadd_ (A_UT_ at which the additional ATP usage appears when P_i_ reaches Pi_crit_), A_UTcrit_ (corresponding to CP, above which no steady-state in the system can be reached) and A_UTsev_ (beginning of S domain), respectively.

## Data Availability

The complete model description is located on the web site: http://bernardkorzeniewski.pl and in the data base BioModels: MODEL2203310001.

## Abbreviations

A_UT_: ATP usage activity
A_UTadd_: A_UT_ at which the additional ATP usage is initiated, M/H border
A_UTcrit_: critical A_UT_, analogous to CP, H/VH border
A_UTsev_: A_UT_ avove which S appears, VH/Sborder
CK: creatine kinaseA
CP: critical power
ESA: each-step activation
GET: gas exchange threshold
H: heavy exercise intensity domain
H space: H/VH border - M/H border
LT: lactate threshold
M: moderate exercise intensity domain
MLSS: maximal lactate steady-state
OXPHOS: oxidative phosphorylation
Pi_crit_: critical P_i_, above which the additional ATP usage is initiated
Pi_peak_: peak P_i_, at which exercise is terminated because of fatigue
PO: power output
S: severe exercise intensity domain
t_0.63_: time to reach 63% of the response amplitude (or the time-constant of an exponential)
VH: very heavy exercise intensity domain
VH space: VH/S border - H/VH border
V̇O_2_: oxygen consumption
V̇O_2__max_: maximal V̇O_2_
